# The utility of pocket-sized echocardiography to assess left ventricular systolic function prior to permanent pacemaker implantation

**DOI:** 10.1186/s12947-015-0004-9

**Published:** 2015-03-14

**Authors:** Lawrence Lau, Robin Ducas, Jacques Rizkallah, Davinder S Jassal, Colette M Seifer

**Affiliations:** College of Medicine, Faculty of Health Sciences, University of Manitoba, Winnipeg, Canada; Section of Cardiology, Department of Internal Medicine, College of Medicine, Faculty of Health Sciences, University of Manitoba, Winnipeg, Canada; Section of Cardiology, Department of Internal Medicine and Associate Chief of Cardiology, Cardiac Sciences Program, Y3019 St Boniface Hospital, Winnipeg, MB UK

**Keywords:** Permanent pacemaker, Pocket-sized echo (PSE)

## Abstract

**Background:**

A subset of patients receiving first-time permanent pacemakers (PPM) may also benefit from an implantable cardioverter defibrillator (ICD) based on the presence of left ventricular systolic dysfunction (LVSD). Routine screening using pocket-sized echocardiography (PSE) may be useful in identifying such patients.

**Objective:**

To determine whether PSE can be used by an inexperienced sonographer to adequately screen for LVSD in a patient population receiving a first-time PPM.

**Methods:**

A sonographic trainee (medical student) acquired images using PSE, which were then evaluated by an experienced echocardiologist for both image quality and presence of LVSD. The sensitivity and specificity of assessment by the inexperienced sonographer was compared to the level 3 echocardiologist.

**Results:**

The patient population included 71 individuals (66% male, mean age 77 ± 12 years). Interpretable images where left ventricular ejection fraction (LVEF) could be adequately assessed were obtained in 93% of the patient population. As compared with the echocardiologist, the sonographic trainee had a sensitivity of 60% and a specificity of 98% in detecting LVSD.

**Conclusions:**

For patients receiving first-time PPM, the use of PSE by a sonographic trainee combined with interpretation by an experienced imaging cardiologist can triage for the need to perform standard transthoracic echocardiography (sTTE) by determining the presence of LVSD.

## Introduction

Permanent pacemakers (PPM) are established therapy for patients with bradyarrhythmias due predominantly to sinus node dysfunction or atrioventricular block [1]. The majority of these patients present with a combination of symptoms including syncope, pre-syncope, fatigue, palpitations, dyspnea, and exercise intolerance. Clinical assessment including a complete history, physical examination, and electrocardiogram or other cardiac rhythm monitoring device usually determines the association between these symptoms and the underlying conduction abnormality. Within the population receiving PPM, there is a subset of patients with structural heart disease who have an underlying predisposition to sudden cardiac death (SCD) due to LVSD that may elude detection by standard clinical assessment. Potential etiologies include coronary artery disease, dilated cardiomyopathy, hypertrophic cardiomyopathy, arrhythmogenic right ventricular dysplasia, or infiltrative cardiomyopathy.

The finding of structural heart disease, in particular LVSD, is pertinent in patients receiving PPM because these patients may have indications for an implantable cardioverter defibrillator (ICD) for prevention of ventricular tachyarrhythmia death, in addition to their pacemaker indication. Several trials have demonstrated reduced mortality associated with implanting an ICD as primary prevention against sudden cardiac death in patients with LVSD in the setting of both ischemic and nonischemic heart disease [2,3]. Accordingly, current practice guidelines recommend ICD implantation for patients with a left ventricular ejection fraction (LVEF) of less than 30-5%, despite optimal medical therapy and, where appropriate, revascularization [4,5].

Although it is reasonable for patients to receive a standard transthoracic echocardiogram (sTTE) to rule out structural heart disease in the context of PPM implantation the relatively resource intensive nature of the sTTE may preclude routine evaluation for all patients undergoing PPM implantation.

Pocket-sized echocardiography (PSE) is a tool that can support routine sTTE evaluation [6]. PSE, a miniaturized version of the sTTE machine, has the capacity for two-dimensional conventional echocardiography and color Doppler. Its size confers the advantage of portability, making PSE ideal for point-of-care evaluation, either at the bedside, or in the outpatient setting. Previous studies have examined the diagnostic accuracy of PSE, its potential clinical applications, and the training involved to maximize safety and effectiveness of its use [7-11]. Several studies have reported that PSE can qualitatively assess LVEF with good reliability, even when a sonographic trainee acquires and/or interprets images [12-18]. Current recommendations limit the use of PSE to triaging candidates to receive sTTE evaluation [19].

Although PSE is not currently routinely used prior to the implantation of PPM, growing interest in its application warrants formal evaluation of its use in this setting. PSE can screen for LVSD in this patient population and determine candidacy for sTTE evaluation, thereby ensuring that all patients undergoing implantation receive the appropriate device. Currently, the need for, and logistics of, performing PSE on this population of patients have yet to be described. This study aimed to determine if left ventricular systolic function could be adequately assessed by a sonographic trainee using PSE, in patients receiving first-time PPM implantation.

## Methods

A prospective study of adult patients undergoing first-time PPM insertion was conducted at a university-affiliated tertiary care hospital between 2012 and 2013. Patients were included if they were ≥18 years of age, had an indication for permanent pacemaker insertion, and consented to participate. Informed consent was obtained from all eligible patients. Exclusion criteria were patients under 18 years of age, undergoing revision of an existing pacing system, or unable to provide informed consent. Ethics approval was granted by the University of Manitoba Biomedical Research Ethics Board (BREB) [B2012:034] and the St Boniface Hospital institutional review committee.

### Training in echocardiography

Prior to recruiting patients for the study, a first-year medical student with no previous background in ultrasound (i.e., “sonographic trainee”) prepared for one month in obtaining and interpreting echocardiographic images. Visual estimations of LVEF on PSE were compared to the LVEF quantitatively determined using biplane Simpson’s on sTTE. During the training period, a total of 80 scans were completed with both PSE and sTTE. The sonographic trainee also interpreted an additional 150 images previously assessed by a level 3 echocardiologist to improve accuracy of visual estimation of LVEF.

### Image and data acquisition

Patients were approached by the sonographic trainee in the pre- and post-cardiac procedure area within two hours of PPM implantation. Immediately after providing informed consent, patients were scanned by the sonographic trainee with PSE. Video loops of parasternal long- and short-axes, apical four- and two-chamber, and subcostal views were obtained and digitally recorded on the PSE device. Relevant medical history was obtained from the patient, hospital charts, and procedural notes.

### Assessment of LVEF

**Figure 1:**
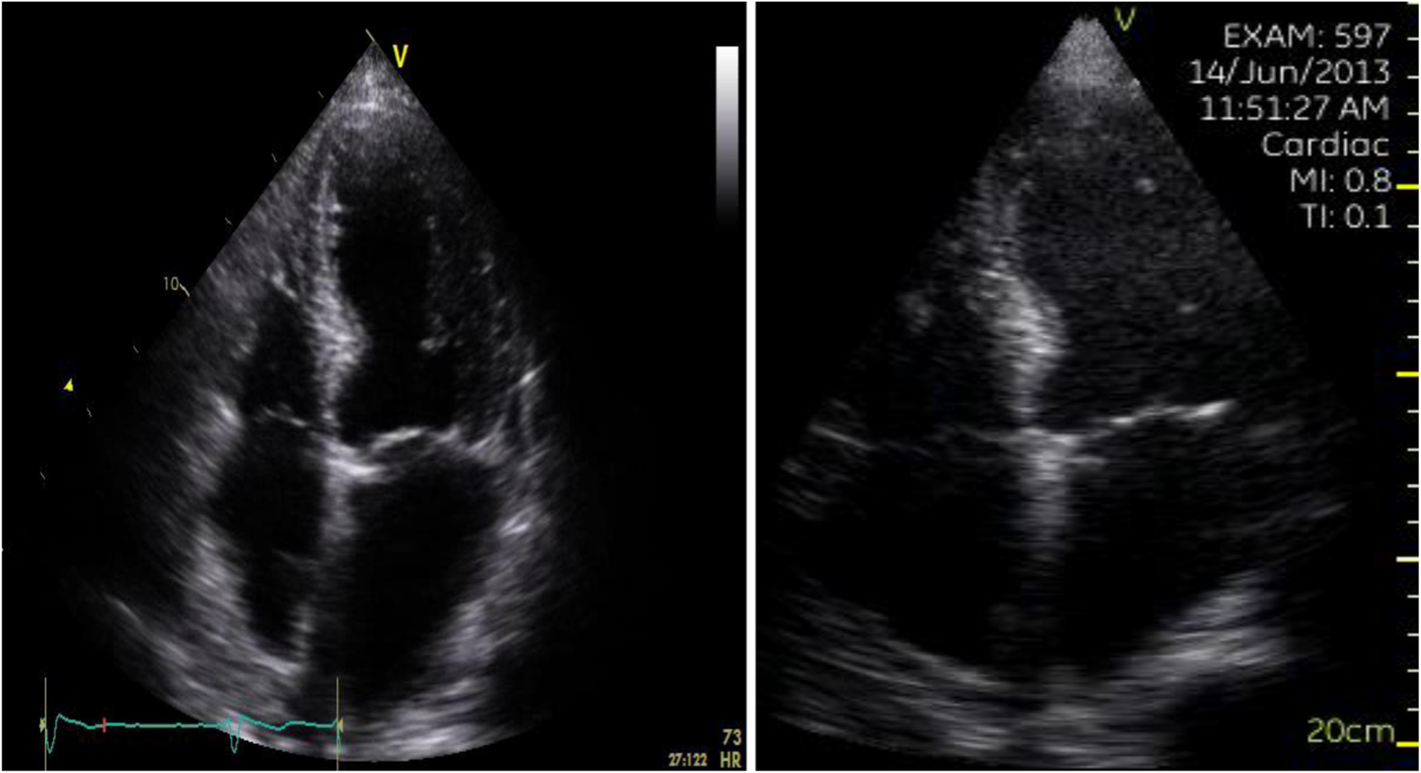
**Comparison of images acquired using (a) sTTE and (b) PSE on the same patient.**

Promptly following PSE scanning, the recorded video loops were reviewed by the sonographic trainee and a cardiologist with level 3 competency in echocardiography, and assessed qualitatively for image quality and LVEF. Figure 1 shows an example of the comparison of images acquired using sTTE and PSE on the same patient. Each interpreter was blinded to the other. Overall LVEF was graded by visual estimation as being normal (≥50%), mildly reduced (40-49%), moderately reduced (30-39%), or severely reduced (<30%). If a significantly reduced LVEF (defined as <40%) was found, the patient was subsequently considered for evaluation with sTTE. If this sTTE examination was consistent with LVSD, the cardiologist implanting the pacemaker was informed.

### Statistical analysis

Statistical analysis was performed with GraphPad Prism 6 (GraphPad Software, Inc., San Diego, CA). Data are presented as percentages and the mean ± SD. The Cohen’s kappa coefficient was used to measure inter-observer concordance for categorical variables, with confidence intervals of 95%. The chi-squared test was used to calculate sensitivity, specificity, positive predictive value, and negative predictive values accordingly.

## Results

### Study population

Of the 75 eligible patients, 71 were enrolled in the study. Four patients were excluded because they declined to provide consent for participation in the study. The baseline clinical characteristics of the study population are summarized in Table 1. The mean age was 77 ± 12 years and 66% were male. Of the total study population, 27 (38%) received a PPM due to sinus node dysfunction, 30 (42%) due to second or third degree atrioventricular block, and 14 (20%) due to a combination of sinus and atrioventricular node dysfunction. Twenty-three patients (32%) were referred for PPM implantation as outpatients, while 48 (68%) were referred from an inpatient unit. The prevalence of presenting symptoms is summarized in Table 2.

**Table 1:** **Baseline clinical characteristics of the study population** ***(n = 71)***

**Characteristic**	**n (%)**
Age (years) ± SD	77 ± 12
Male	47 (66)
Indication for PPM implantation	
Sinus node dysfunction (SND)	27 (38)
Atrioventricular nodal block (AVB)	30 (42)
Both SND and AVB	14 (20)
Atrial fibrillation	28 (39)
Smoking history (>10 pack years)	28 (39)
Hypertension	52 (73)
Diabetes mellitus type 2	16 (23)
Dyslipidemia	22 (31)
Coronary artery disease	20 (28)
Previous myocardial infarction	19 (27)
Previous stroke or transient ischemic attack	13 (18)
History of congestive heart failure	15 (21)

**Table 2:** **Symptoms at initial patient presentation to health care centre** ***(n = 71)***

**Presenting symptom**	**n (%)**
Syncope	25 (35)
Presyncope	43 (61)
Fatigue	23 (32)
Exercise intolerance	11 (15)
Dyspnea	26 (37)
Palpitations	9 (13)
Edema	10 (14)

### PSE image acquisition and interpretation

PSE video clips were obtained and recorded for all enrolled participants. The mean time required to perform the scan was 5.8 ± 2.6 minutes (range: 2–15 minutes). Image quality was assessed as “good” in 40%, “fair” in 51%, and “poor” in 9%. Overall, LVEF could be reliably assessed from PSE images in 66 of the 71 patients (93%). The distribution of LVEF in the study population as assessed by PSE is summarized in Table 3. Of these interpretable images, there was 91% concordance between the sonographic trainee and echocardiologist in the assessment of LVEF, with a linearly weighted Cohen kappa value for inter-observer concordance of 0.725 ± 0.112 (95% CI 0.505-0.945). As compared to the echocardiologist, LVEF was overestimated in 5% and underestimated in 5% by the sonographic trainee. The inter-observer concordance between the sonographic trainee and echocardiologist was 96% for the ability to assess presence or absence of LVSD. (Table 4) Conversely, the sonographic trainee falsely identified the presence of LVSD in one (2%) scan, while missing the presence of LVSD in two (3%) scans. The kappa value for inter-observer variability in the assessment of LVSD was 0.643 ± 0.201 (95% CI 0.247-1.0). The sensitivity and specificity of the sonographic trainee’s evaluation compared to the cardiologist’s evaluation for LVSD was 60% and 98%, respectively.

**Table 3:** **LV ejection fraction based on estimation of PSE images** ***(n = 71)***

**LV ejection fraction**	**Sonographic Trainee n (%)**	**Echocardiologist n (%)**
Normal (>50%)	60 (85%)	58 (82%)
Mild dysfunction (40-50%)	6 (8%)	3 (4%)
Moderate dysfunction (30-40%)	2 (3%)	2 (3%)
Severe dysfunction (<30%)	2 (3%)	3 (4%)
Uninterpretable	1 (1%)	5 (7%)

**Table 4:** **Assessment of PSE images for presence or absence of significant LVSD by sonographic trainee vs. echocardiologist** ***(n = 66)***

		**Echocardiologist evaluation**	
		**LVSD absent (LVEF > 40** **%** **)**	**LVSD present (LVEF < 40** **%** **)**
**Sonographic trainee interpretation**	LVSD absent (LVEF > 40%)	60 (91%)	2 (3%)
	LVSD present (LVEF < 40%)	1 (1%)	3 (5%)

### Accuracy of PSE compared to sTTE

Of the total study population, 28 patients (39%) received a sTTE for a clinical indication within 12 months of inclusion in this study. We took this opportunity to compare these images with those obtained by PSE. Of these patients, 2 (7%) had PSE exams that were not interpretable. For the remaining 26 patients, the accuracy of LVEF estimation by PSE as compared to sTTE was 96% for the echocardiologist and 88% for the sonographic trainee. The echocardiologist correctly assessed 100% of patients for the presence or absence of LVSD (LVEF either greater or less than 40%) using PSE images, whereas the sonographic trainee correctly assessed 96%.

## Discussion

With advancements in cardiovascular imaging, it is tempting to seek out new clinical applications to justify new technology. While individual patients may benefit from increased access to diagnostic tools, formal evaluation of the overall feasibility of this tool is necessary prior to routine institution. The results of this study suggest that PSE can identify patients receiving first-time PPM at risk of SCD due to LVSD.

The use of the PSE to visualize left ventricular wall motion and systolic function has been previously evaluated. Several studies have demonstrated that the PSE can obtain interpretable images in the vast majority (85-100%) of patients by inexperienced sonographers, in both the inpatient and outpatient settings [10-12]. A complete sTTE study takes approximately 20–25 minutes to acquire, with a standard echocardiography machine costing approximately four times that of a PSE machine [20]. The present study demonstrated that the feasibility (94% of images were interpretable) and speed (average time to scan of 6 minutes) of PSE image acquisition were suitable for pre-implant echocardiographic evaluation.

Previous studies comparing images acquired by an inexperienced sonographer (e.g., medical resident) versus an experienced sonographer (e.g., cardiologist with level 3 competency in echocardiography) have shown that there is good correlation between PSE and sTTE for the assessment of LVEF [12-18]. High inter-observer correlation between the trainee and the imaging cardiologist is important in order to appropriately triage patients for sTTE in the setting of pre-PPM implantation. This study found good concordance (κ = 0.725) in categorizing LVEF into normal, mildly reduced, moderately reduced, and severely reduced. This was similar to the concordance (κ = 0.606) rate described by Panoulas and colleagues, although in their study medical students and the cardiologist evaluated separately acquired images [18]. Compared to the evaluation by the echocardiologist, the sonographic trainee had a good specificity (98%) but a poor sensitivity (60%), reflecting a tendency to “over-call” LVSD. This suggests that a sonographic trainee can assess PSE images for LVSD with good concordance compared with an echocardiologist in order to triage candidates for a more sensitive sTTE evaluation.

PSE studies show that the strength of correlation with sTTE is dependent upon level of training in echocardiography, which differ depending on patient setting [15-18,21]. No studies have examined the accuracy of PSE in a pre-PPM implant setting. Although this study did not formally compare PSE directly with sTTE, 28 of the 71 patients underwent sTTE as part of their clinical assessment. LVEF as estimated from PSE images by the echocardiologist compared to sTTE were concordant in 96% of these 28 patients. As expected, the sonographic trainee was slightly less accurate in describing LVEF from PSE images, with 88% concordance. There was greater success in detecting LVSD than estimating LVEF; the echocardiologist was able to identify the presence or absence of LVSD in all (100%) patients, and the sonographic trainee was able to do so in all but one patient (96%). Overall, the evaluation of LVEF with PSE in the PPM pre-implant population was comparable to that of sTTE in this subgroup of patients.

This study provides insight into how PSE may be implemented into the pre-procedural assessment prior to the insertion of a PPM. There are few published studies describing the pre-procedural or pre-operative role of PSE. Frederiksen and colleagues demonstrated that PSE can be used for pre-operative assessment for day surgery patients and determined that the parasternal long axis view alone, taken with the patient in a sitting position, suffices to estimate LVEF [7]. In contrast, all participants in this study were scanned with PSE in a supine position, which enabled a comprehensive echocardiographic exam amenable to enhancing maneuvers, such as lying in the left lateral decubitus position.

### Limitations and future directions

This study presented several limitations. First, as a pilot study, the number of patients included was small, and accordingly, was not intended to affect clinical management of patients undergoing PPM implantation who were enrolled as participants. Although the cardiologist implanting the pacemaker was notified of the presence of possible LVSD on PSE, a protocol for management of LVSD found on PSE was not formally instituted. Although this study commented on the accuracy of PSE compared to sTTE in a subset of patients, this was not the primary objective. Image accuracy has been previously well described in various other patient populations [7-18]. Finally, although this study validated the use of PSE in assessing LVEF, if more extensive information on cardiac structure and function is required clinically prior to PPM implantation sTTE is superior to that of PSE. For example, other cardiomyopathies including hypertrophic cardiomyopathy, noncompaction of the left ventricle, and arrhythmogenic right ventricular dysplasia may present with bradyarrhythmias and a high risk of SCD due to tachyarrhythmias. It follows that there are limitations in training a sonographer only to interpret LVEF; broader training with PSE in other aspects of echocardiography may be important to provide more extensive use of this technology.

## Conclusions

PSE is a bedside tool that can effectively screen for LVSD in patients undergoing PPM. An inexperienced sonographic trainee can learn to acquire images quickly and accurately resulting in minimal disruption of the existing pre-procedure workflow. This technology may assist in identifying patients who require more complex cardiac implantable electronic devices.
